# Wearable sensors can reliably quantify gait alterations associated with disability in people with progressive multiple sclerosis in a clinical setting

**DOI:** 10.1007/s00415-020-09928-8

**Published:** 2020-05-28

**Authors:** Lorenza Angelini, William Hodgkinson, Craig Smith, Jessie Moorman Dodd, Basil Sharrack, Claudia Mazzà, David Paling

**Affiliations:** 1 Department of Mechanical Engineering and Insigneo Institute for in silico Medicine, University of Sheffield, Pam Liversidge Building, Mappin Street, Sheffield, S1 3JD UK; 2 Medical School, University of Sheffield, Sheffield, UK; 3 Academic Department of Neuroscience, Sheffield NIHR Neuroscience BRC, Sheffield Teaching Hospital NHS Foundation Trust, Sheffield, UK; 4 Sheffield Institute of Translational Neuroscience, Sheffield Teaching Hospital NHS Foundation Trust, Sheffield, UK

**Keywords:** Test-retest reliability, Gait analysis, Balance, Temporal parameters, Regularity, Six-minute walk

## Abstract

Gait disability in people with progressive multiple sclerosis (MS) is difficult to quantify using existing clinical tools. This study aims to identify reliable and objective gait-based biomarkers to monitor progressive multiple sclerosis (MS) in clinical settings. During routine clinical visits, 57 people with secondary progressive MS and 24 healthy controls walked for 6 minutes wearing three inertial motion sensors. Fifteen gait measures were computed from the sensor data and tested for between-session reliability, for differences between controls and people with moderate and severe MS disability, and for correlation with Expanded Disability Status Scale (EDSS) scores. The majority of gait measures showed good to excellent between-session reliability when assessed in a subgroup of 23 healthy controls and 25 people with MS. These measures showed that people with MS walked with significantly longer step and stride durations, reduced step and stride regularity, and experienced difficulties in controlling and maintaining a stable walk when compared to controls. These abnormalities significantly increased in people with a higher level of disability and correlated with their EDSS scores. Reliable and objective gait-based biomarkers using wearable sensors have been identified. These biomarkers may allow clinicians to quantify clinically relevant alterations in gait in people with progressive MS within the context of regular clinical visits.

**Electronic supplementary material:**

The online version of this article (10.1007/s00415-020-09928-8) contains supplementary material, which is available to authorized users.

## Introduction

Multiple sclerosis (MS) is a common immune-mediated inflammatory and degenerative disease of the brain and spinal cord [1]. The initial clinical course is variable, but the majority of patients either present with or transition into a progressive course, characterised by the gradual accumulation of disability independent of clinical relapses, which usually significantly affect their ability to walk. It is estimated that more than 1.3 million people have progressive MS worldwide. Currently, few disease-modifying therapies are available for this phase of the illness [1, 2].

Walking related disability has a significant impact on the quality of life [3] and is rated by people with MS as one of their worst symptoms [4]. Despite its importance, it is difficult to quantify this disability within clinical and research settings [5]. Typical assessments include clinical evaluation, rating scales (e.g., Expanded Disability Status Scale (EDSS) [6], 12-item MS Walking Scale [7]), and timed or distance tests (e.g., Timed 25-foot Walk, 10-m Timed Walk, 30-m Timed Walk, 100-m Timed Walk, and 2-min or 6-min Walk Test [8]). Whilst EDSS and other composite endpoints, such as the MS Functional Composite [9], have been used in clinical trials in progressive MS, these outcome measures are insensitive to the small alterations in walking disability that accumulate over the time course of a clinical trial [10]. More sensitive measures would enable clinicians to identify people with objective evidence of progressive MS more confidently, help with clinical decisions related to prognosis and the use of disease-modifying therapies, and could serve as biomarkers of disease progression in clinical trials [1, 11].

In recent years there has been increasing interest in body*-*worn technology for quantification of disease-related gait changes [12–18]. However, their use as part of the clinical pathway of people with progressive MS is still very limited. This is partly due to the fact that previous studies had significant heterogeneity, both from the clinical and methodological perspective, and did not have a specific focus on progressive MS, which made it unclear which set of gait measures might be able to discriminate and predict different levels of walking-related disability in these people. Gait is a complex sensorimotor activity that involves not only the spatial and temporal coordination of the lower limbs but also the coordination of the trunk and arms as well as the dynamic balance [19]. Although the latter factors are known to be affected in people with MS [20], the majority of previous studies only looked at a limited subset of gait measures based on spatio-temporal features (e.g., step or stride length, step or stride duration, and gait speed, etc.), which might allow capturing only few components of disease*-*specific gait impairments [21]. To broaden the scope of the assessment, gait measures like intensity, jerk, regularity, and symmetry have been proposed to characterize the overall “quality and energetic efficiency” of an individual’s gait [22]. These have been successfully applied to both older adults [23], those at risk of falling [24] or affected by neurological disorders such as Parkinson’s disease, where they have been found to add valuable and complementary information to traditional gait analysis [21]. In MS, the use of this approach has been limited to understanding the effects of fatigue [18] and gait changes in the real world [25], but the feasibility of employing such gait measures as tools for quantifying gait abnormalities in people affected by this condition and for integrating them into routine clinical assessments is yet to be investigated. The present study is, therefore, designed to fill in this gap.

This study sought to identify biomarkers that could allow reliable and objective characterisation of gait alterations in people with progressive MS with different levels of disability compared to healthy controls, using sensors worn on the shins and lower back whist performing a walking test during routine outpatient visits. To this end, we aimed to: (1) assess between*-*session reliability of a comprehensive set of gait measures in healthy adults and in people with progressive MS and (2) determine which gait measures could discriminate between people with progressive MS with different levels of disability in a clinical setting.

## Methods

### Participants

Fifty*-*seven people with secondary progressive MS and 24 healthy controls took part in this study (Table 1). Participants were recruited either from the Sheffield MS Clinic at the Royal Hallamshire Hospital (Sheffield, United Kingdom) when they attended for their routine appointments or from the Sheffield Clinical trial Unit where a cohort of people with secondary progressive MS taking part in a double-blinded intervention-based clinical trial [26] attended for their baseline assessment. None of the patients had a relapse or change of medication in the previous 3 months and none was recovering from an infection or an intercurrent illness. None of the healthy controls had any other medical or orthopaedic condition affecting their walking.

**Table 1:** Demographic and clinical characteristics of the study participants

	People with MS	Healthy controls	Statistics		
			Ctrl vs MSm	Ctrl vs MSs	MSm vs MSs
*N* subjects	MS: 57				
	MSm: 25	24			
	MSs: 32				
Age [years]	MS: 56.0 (9.3)				
	MSm: 55.8 (8.2)	49.8 (8.4)	*t*(47) = − 2.51, *p* = 0.02*	*t*(54) = − 2.48, *p* = 0.02*	*t*(55) = − 0.16, *p *= 0.87
	MSs: 56.2 (10.2)				
Gender [% female]	MS: 67.8%				
	MSm: 65.4%	66.7%	*χ*^2^(1) = 0.04, *p* = 0.85	*χ*^2^(1) = 0.18, *p* = 0.68	*χ*^2^(1) = 0.40, *p* = 0.53
	MSs: 69.7%				
EDSS scores	MS: 5.5 (3.0–6.5)				
	MSm: 4.0 (3.0–5.0)				*U* = 0.00,* z* = − 6.55,* p* < 0.001
	MSs: 6.0 (5.5–6.5)				
Walking assistive device [%]	Without: 61%				
	Unilateral: 25%				
	Bilateral: 14%				

Data are represented as mean (standard deviation) if normally distributed or as median (range) if not normally distributed. *EDSS* Expanded Disability Status Scale, *Ctrl* healthy controls

*The 6- and 7-year average age difference between Ctrl and MSm and Ctrl and MSs, respectively, is not expected to affect gait measures within this age range [44].

Disability was assessed using the EDSS and people with MS were split into two subgroups around the median EDSS score. People with EDSS score between 3 and 5 were classified as being moderately disabled (moderate MS, MSm) whereas people with EDSS score higher than 5 were classified as being severely disabled (severe MS, MSs).

### Experimental procedure

**Fig. 1:**
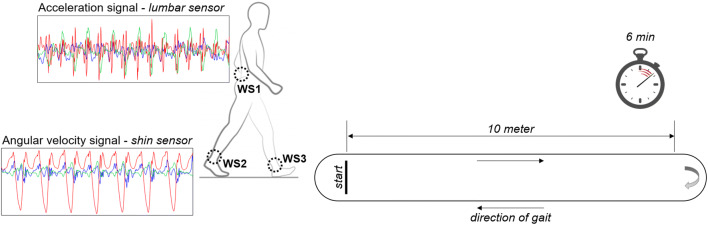
Gait protocol and positioning of the wearable sensors (WS1–3). Acceleration and angular velocity signals were recorded during the walking test using three wearable sensors placed on the anterior shins and on the lower back. Typical raw acceleration and angular velocity data recorded over time along anterior-posterior (AP, green line), medio-lateral (ML, red line), and vertical (V, blue line) axes are shown on the left

Participants were fitted with three tri-axial inertial sensors (OPAL, APDM Inc., Portland, OR, USA) using adjustable Velcro straps (Fig. 1). Two of these were attached on the anterior shin on the right and left with the aim to accurately estimate the temporal measures (e.g., step or stride duration) [27], and one was placed on the back overlying the fifth lumbar vertebra [15] to extract the gait quality measures related to poor balance control and altered coordination [20]. Acceleration and angular velocity signals were recorded along the anatomical vertical, medio-lateral, and anterior-posterior axes at a sampling frequency of 128 Hz and the accelerometer range was set at ± 6 g.

After being fitted with the sensors, participants were asked to walk back and forth in a hospital corridor for 6 minutes (Fig. 1). Since the test was not meant to quantify the submaximal level of functional capacity and since we aimed to propose a solution that could be widely adopted in standard hospital settings, the length of the path was limited to 10 m, with adequate space for turning around at each end. Participants were asked to walk at their comfortable speed and could rest and/or to use assistive devices if needed. No verbal interaction with other people was allowed during testing.

In order to assess between*-*session reliability, 11 people with MSm, 14 people with MSs, and 23 healthy controls repeated the assessment on a second visit, which was held 7–14 days after the first one. The sample size calculation was based on the previous work by Zou [28] and considered an expected ICC value of 0.85 with an acceptable lower limit of 0.40, a power of 80%, and a confidence interval of 95%. Participants were assessed at the same time of the day, and patients had not experienced a relapse, change in medication, or intercurrent infection between the two assessments.

### Sensor data processing

**Fig. 2:**
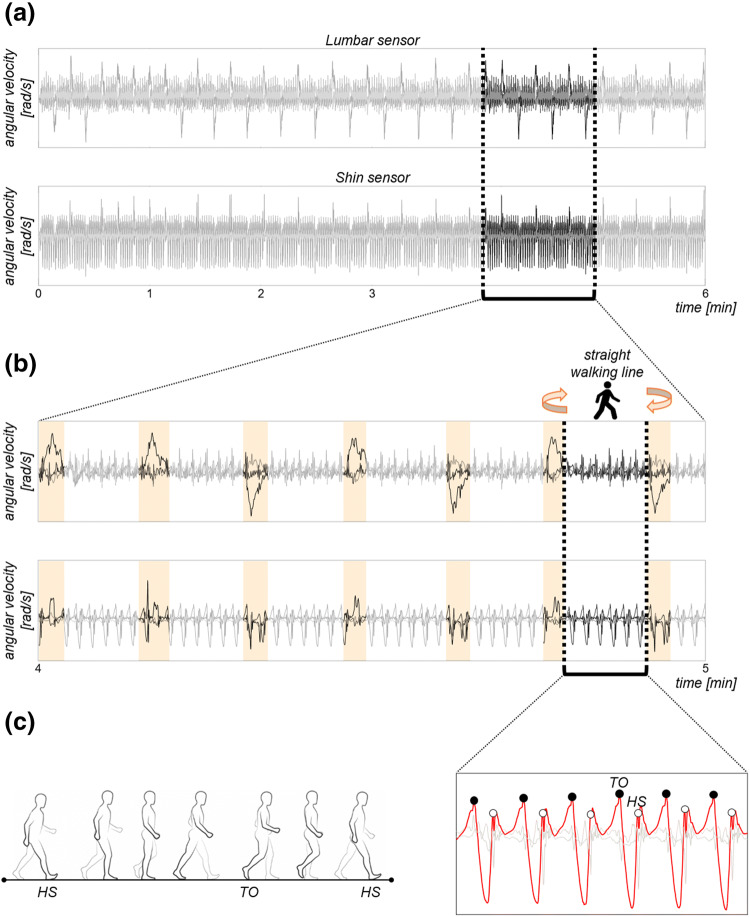
Sensor data processing. **a** Example of angular velocity signals recorded using the lumbar and shin sensors during the walking test. **b** Zoom in on angular velocity signals between minute 4 and minute 5. Identification of the straight walking lines and removal of the turning times (light orange bars) from the signals. **c** Zoom in on one straight walking line. Detection of initial contacts (heel strike, HS, white circles) and final contacts (Toe-Off, TO, black circles) of each foot with the floor. HS occurs when heel comes in contact with the floor, while TO when toe is off the floor. HS and TO events were identified for each walking pass by finding peaks in the angular velocity of the shin sensors along the medio-lateral axis (ML, red line)

Figure 2 summarizes the main steps involved in the analysis of the sensor data (i.e., acceleration and angular velocity signals) collected during the walking test.

First, the tri*-*axial accelerations were reoriented to a horizontal-vertical coordinate system and filtered with a 10 Hz cut-off, zero phase, low*-*pass Butterworth filter [29]. Second, accelerations and angular velocities over 6 minutes (Fig. 2a) were segmented into walking passes (i.e., straight walking lines), with turning and resting times detected from the lumbar angular velocities [30] and discarded from subsequent analysis (Fig. 2b). Only steady-state walking passes were, therefore, processed to compute the gait measures of interest. Finally, initial floor contacts and final floor contacts of each foot were identified for each walking pass by searching for local maxima in the shin medio*-*lateral angular velocity of both legs (Fig. 2c) [31]. The initial contacts were referred to as Heel*-*Strike (i.e., the moment when the heel strikes the floor, HS), while the final contacts were referred to as Toe*-*Off (i.e., the moment when the toe leaves the floor, TO).

### Gait measures

The sensor data were processed into 15 gait measures, grouped into three domains: rhythm, variability, and balance and coordination [21, 32].

*Rhythm* Stride, step, stance, swing, single support, and double support durations were selected to represent the rhythm domain (Table 2). These gait measures were computed based on the timing of HS and TO events (see Fig. 2c).

**Table 2:**
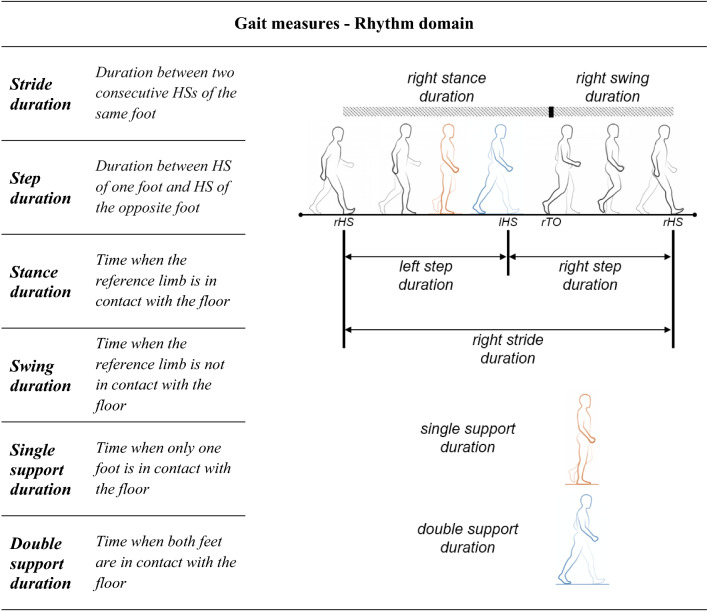
Rhythm domain measures

*rHS *right heel strike, *lHS* left heel strike, *rTO* right toe-off

**Table 3:**
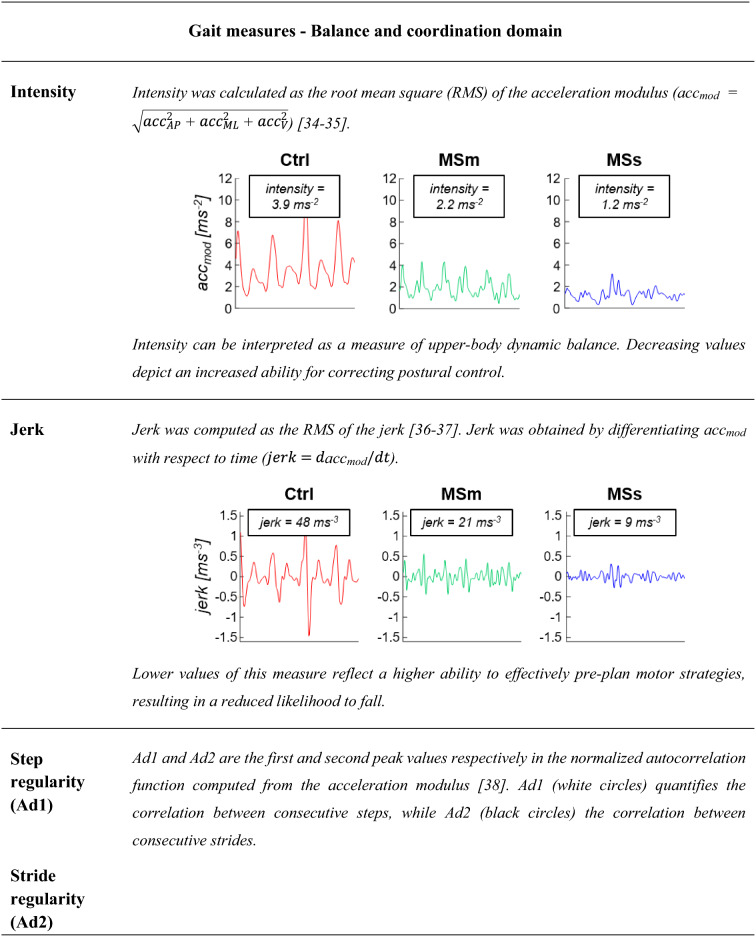

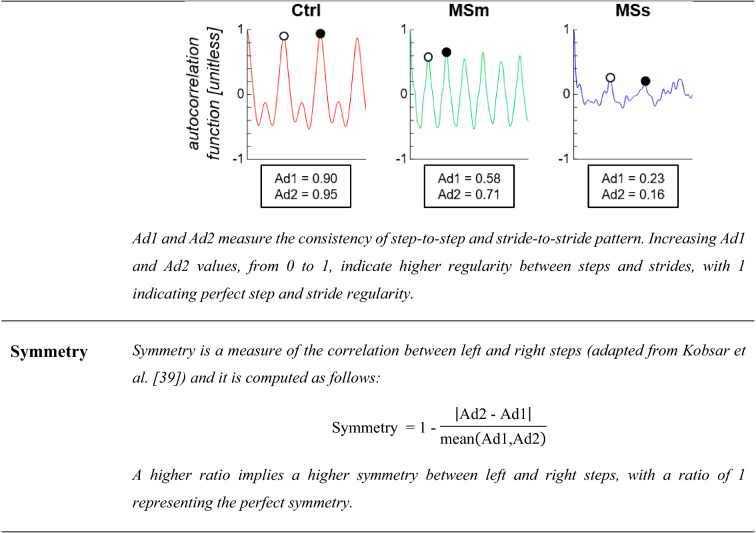
Balance and coordination domain measures

Differences in intensity, jerk, step regularity, stride regularity, and symmetry are shown for a representative healthy subject (Ctrl, red line), a representative person with moderate MS (MSm, green line), and a representative person with severe MS (MSs, blue lines).

*AP* anterior-posterior, *ML* medio-lateral, *V* vertical

**Fig. 3:**
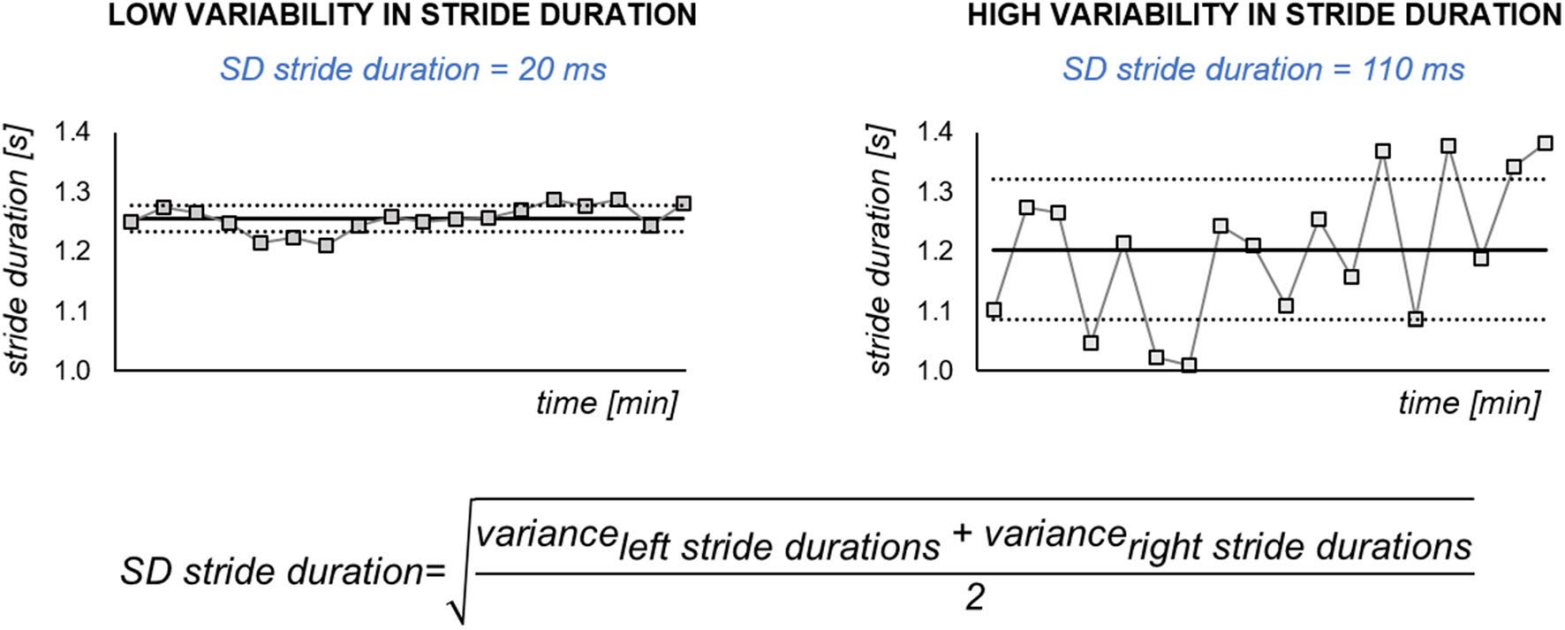
Variability domain measures. Example of low and high variability (i.e., standard deviation (SD)) in stride duration

*Variability* The variability in stride, step, stance, and swing durations were calculated, including at least 50 steps as described in Galna et al. [33]. For example, variability in stride duration was defined as the combined within-person standard deviation of the left and right stride durations (Fig. 3).

*Balance and coordination* Intensity, jerk, step regularity, stride regularity, and symmetry were computed from the lumbar acceleration signals for each walking pass with a minimum of five consecutive strides.

Table 3 provides a description and a visual representation of each gait measure used to characterize the gait of people with MS and healthy controls.

### Statistical analysis

Participant characteristics were compared using Pearson’s Chi-square for gender and independent Mann-Whitney U for age and EDSS scores. For each participant, all the gait measures were averaged over the walking time.

Between-session reliability was evaluated for participants who completed two visits. Intraclass Correlation Coefficients (ICCs) were calculated using the ICC(2,k) model with 95% confidence intervals [40]. Thresholds for ICC values were defined as per guidelines from Li et al. [40]: 0.75 or higher indicates excellent reliability, 0.6–0.74 indicates good reliability, 0.4–0.59 indicates fair reliability, and ICC lower than 0.39 indicates poor reliability. The Bland-Altman analysis was also performed to assess the agreement between the sets of gait measures obtained in the two visits [41].

Correlation between each gait measure and the EDSS score was assessed in the MS group using a Spearman’s rank correlation coefficient (r).

The non-parametric Kruskal test was performed to compare gait measures from the control, MSs and MSm groups since a Shapiro-Wilk test showed that the gait measures were not normally distributed. Where a statistically significant difference was found (*p-*value < 0.05), an independent post hoc test (Mann-Whitney *U* Test) with Bonferroni correction was carried out at a 1% level of significance to accommodate for multiple comparisons (i.e., (i) people with MSm vs healthy controls, (ii) people with MSs vs healthy controls, (iii) people with MSm vs people with MSs). Median, median absolute deviation (MAD) and range values were calculated across participants for each of the investigated gait measures.

The gait measures for people with MSm and people with MSs were normalized with respect to those for healthy controls (ctrl) by calculating the robust *z*-scores (*z*_r_, [42]) as follows:

$$z_{{\text{r}}} = \frac{{{\text{median(gait measure}}_{{{\text{people with MS}}}})-{\text{median(gait measure}}_{{{\text{ctrl}}}})}}{{{\text{MAD(gait measure}}_{{{\text{ctrl}}}})}},$$

where $$\text{MAD(gait measure}_{{{\text{ctrl}}}})=1.4826 \times\text{median(|gait measure}_{{{\text{ctrl(i)}}}}-\text{median(gait measure}_{{{\text{ctrl}}}})|)$$.

Radar plot, including the *z*_r_ *-*score values, were used to give an overview of all the investigated gait measures and to highlight the strength of each gait measure in distinguishing people with MS with different levels of disability with respect to healthy controls. The central line in the radar plot represents healthy controls (*z*_r_ -score = 0) and deviation from zero along the radial axes indicates how people with MSm and people with MSs differ from controls.

The effect size (d) for non-parametric tests was also computed as follows:

$$d = \frac{z}{{\sqrt {\text{N}} }},$$

where *z* is the *z*-score, and N is the number of total observations on which *z* is based. Thresholds of 0.1, 0.3, and 0.5 were recommended by Cohen [43] for small, medium, and large effect sizes, respectively.

## Results

### Participant characteristics

Participant characteristics are summarized in Table 1.

### Between-session reliability

**Fig. 4:**
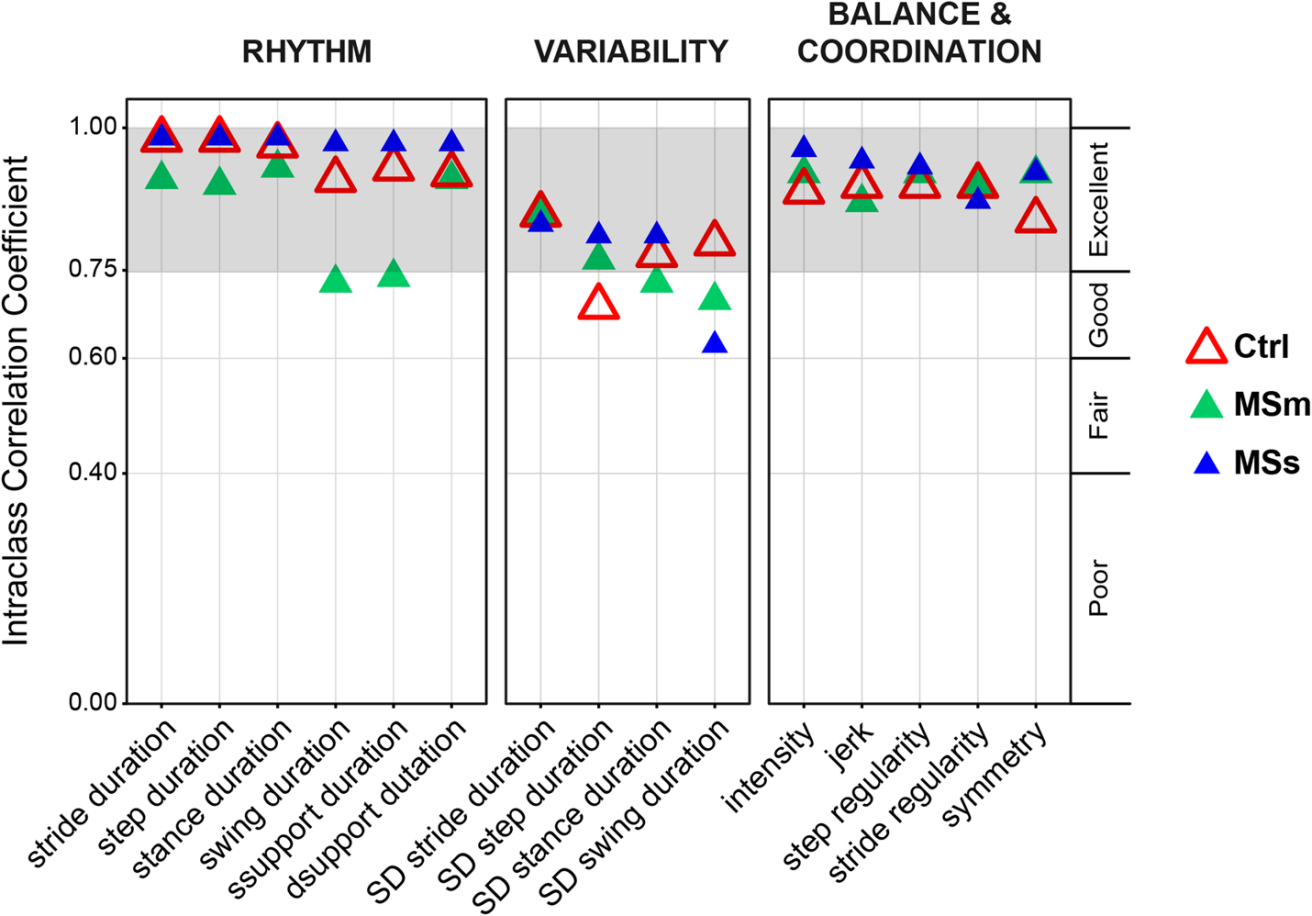
Intraclass correlation coefficients (ICCs). ICCs were calculated for healthy controls (Ctrl, red triangles), people with moderate MS (MSm, green triangles), and people with severe MS (MSs, blue triangles) who completed two testing visits, 7–14 days apart in order to evaluate the between-session reliability of each gait measure. Excellent between-session reliability is depicted in grey colour

Most of the gait measures demonstrated good to excellent between-session reliability (Fig. 4) for healthy controls (mean ± standard deviation ICC: 0.88 ± 0.08), people with MSm (0.85 ± 0.08), and people with MSs (0.90 ± 0.10). The Bland-Altman plots (Supplementary Fig. 1) also showed a good agreement between the sets of gait measures obtained in the two visits.

For healthy controls, 14 out of 15 gait measures showed excellent and only 1 (variability in step duration) good between-session reliability. For people with MSm, 11 out of 15 measures revealed excellent and the remaining 4 good between-session reliability. Finally, for people with MSs, 14 out of 15 measures exhibited excellent and only 1 (variability in swing duration) good between-session reliability.

### Gait measures

**Fig. 5:**
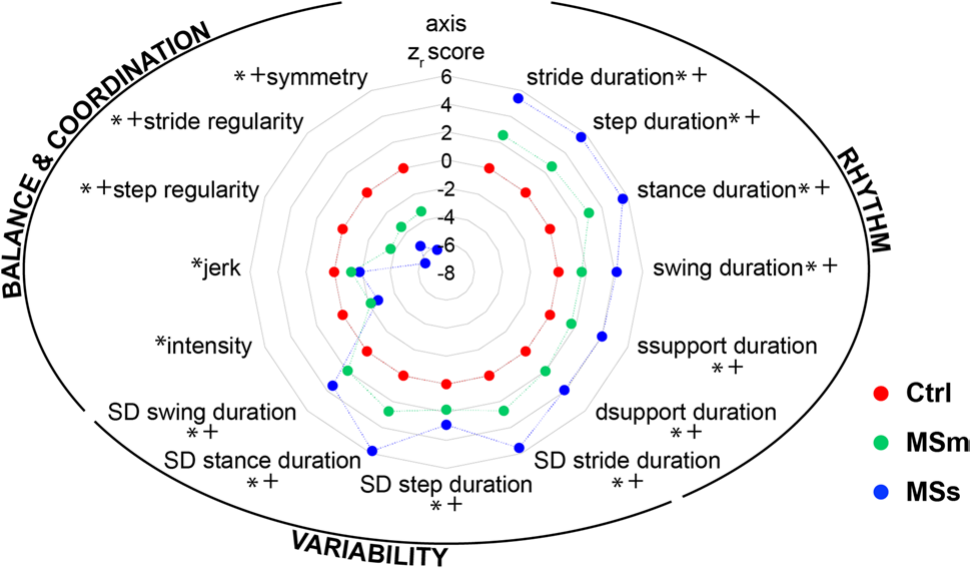
Gait measures representative of rhythm, variability, and balance and coordination domains. Gait measures were calculated for healthy controls (Ctrl, red markers), people with moderate MS (MSm, green markers), and people with severe MS (MSs, blue markers). Note that increasing *z*_r_-score values in this radar plot indicate less rhythmic gait pattern (rhythm domain), more variable gait pattern (variability domain) and less difficulties in controlling balance and coordination (balance and coordination domain). *z*_r_-scores are based on median and median of absolute deviations (MAD) of Ctrl. Each radial line along the axes represents ± 2MAD. Numerical values of median, MAD, and range, together with p-values and associated effect sizes, are reported in the supplementary material (Table 1). *Indicates significant differences between Ctrl and people with MSm and between Ctrl and people with MSs. + Indicates significant difference between people with MSm and people with MSs

Gait measures highlighted significant alteration in gait dynamics both in people with MSm and in people with MSs with respect to healthy controls (i.e., MSm vs healthy controls and MSs vs healthy controls), and between people with different levels of disability (i.e., MSm vs MSs) (Fig. 5).

When compared to healthy controls, gait measures showed that both people with MSm and people with MSs walked with a less rhythmic gait pattern, consisting of significantly longer step, stride, stance, swing, and single and double support durations. These measures also showed significant disruption in the normally strictly timed dynamics of HS and TO events, with increased variability seen in stride, step, stance, and swing durations (Fig. 5). Furthermore, the gait pattern in people with MSs became much less rhythmic and much more variable with respect to that observed in people with MSm (Fig. 5). This was also confirmed by the correlation between the gait measures representative of rhythm and variability domains and EDSS scores with r values ranging between 0.48 and 0.61.

When additional data were leveraged from the lumbar sensor, to give a broader view of the temporal pattern of gait, our results found a reduction in intensity and jerk both in people with MSm and MSs with respect to healthy controls, indicating a higher ability to stabilize their balance with a smoother walking pattern in the presence of an impairment of the lower limbs. Our results were also able to show a decrease in the normal consistency of gait, as detected in lower values for stride regularity, step regularity, and symmetry. These differences were more marked in people with MSs than in those with MSm (Fig. 5), with values of the gait measures being on average three times larger in the former group. Weak to moderate associations (r values between 0.24 and 0.46) were observed between the gait measures representative of balance and coordination and EDSS scores.

## Discussion

Gait is complex, consisting of periodic highly sequenced and conserved movements characterised by rapid contractions and relaxations of muscle groups. By paradox, these rapid movements must propel the body forward with unvarying speed whilst simultaneously being highly energy-efficient and stable.

The pathological changes which affect the central nervous system in MS interferes with the ability of affected individuals to generate these complex movements, with eventual consequences on their walking and stability leading to falls and increasing disability.

The aim of this study was to see if small wearable sensors, integrated into the normal clinical assessment of people with MS during their routine outpatient clinic visits, were able to characterise alterations in gait that occurred in progressive MS accurately and reproducibly. Overall, the results indicated that people with MS walk at a slower pace and with a variable gait pattern of steps and strides, and have difficulties in controlling the movements of their trunk, with such impairments being more evident in people with higher degrees of disability.

Fifteen gait measures were calculated during the instrumented walking test. Almost all gait measures showed good to excellent reliability (ICC > 0.6) across two separate testing days for the three groups (Fig. 4).

This study showed that ICCs in healthy controls were slightly lower than those in people with MS. Similar findings were also found in other studies [45]. A possible explanation is that since walking is not challenging for healthy adults, they often walk using a variety of normal walking patterns. In people with severe MS, some of the variables showed large confidence intervals, indicating that this subset of data might not be suitable for assessing responsiveness.

Most of the gait measures assessed in this study differed significantly between people with MS and healthy controls (Fig. 5). Alteration in the speed of gait was noted in people with MS, with longer stride and step durations, stance and swing durations and single and double support durations. These findings complement the results seen in previous studies in people with MS with moderate and severe levels of disability [14–17, 46].

Alteration in the pattern of gait was also noted, with much greater variability, in the temporal measures in people with MS (Fig. 5). Socie et al. [47] reported similar findings for variability in step duration. We also found significantly more gait variability in people with greater disability. This is consistent with the positive correlation between gait variability and EDSS scores reported by Socie et al. [47]. This higher gait variability could be related to fatigue, decreased muscle strength and impaired balance [47]. Moreover, gait variability could also be a marker of a fear of falling [48].

We also identified alteration in the stability of the upper body as shown by the balance and coordination domain (Fig. 5). Few studies have previously examined measures additionally derived from a sensor on the lumbar spine in people with MS [12, 13, 15, 18]. We found gait intensity to be significantly lower in people with MS compared to controls (Fig. 5). This suggests that people with MS tend to minimize upper body movement to achieve higher stability when walking with reduced leg strength [49, 50]. Similar results are seen in normal ageing [51] and in patients following stroke [52]. In agreement with the findings of studies involving other neurological conditions [21, 36], jerk was also significantly reduced in people with MS (Fig. 5) as a result of compensation strategies to maintain dynamic stability under adverse conditions. Stride and step regularity were found to be the most sensitive measures to discriminate people with MS with different levels of disability from healthy controls. People with MSm showed a significant lower step and stride regularity when compared with healthy controls, and this was increased in people with higher level of disability (i.e., people with MSs) (Fig. 5). This suggests that people with MS are less able to regulate repeating steps and strides during gait and to control the rhythmic displacements of the upper body during walking. Similar results were reported by others in MS [18] and in Parkinson’s disease [21].

A possible limitation of this study, related to our decision to perform the walking test along a 10-m pathway, is the low number of strides (i.e., five strides for each straight walking pass) included for calculating step regularity, stride regularity, and symmetry. The validity of this choice is, however, supported by the existing literature. Tura et al. [53] found that the minimum number of strides needed for reliable computation of step regularity, stride regularity, and symmetry through autocorrelation sequence is between two and four steps recorded at steady-state both in healthy controls and in above-knee amputees. Additionally, Moe-Nilssen, Helbostad [38] suggested that the number of strides needed for this assessment is five in healthy adults. More recently, Angelini et al. [30] showed that, in people with MS, the gait measures we investigated in this manuscript are robust to changes in the experimental procedures, including the length of the walkway. The proposed gait measures could, therefore, be reliably integrated into the assessment performed in any clinical facility where 10-m walk measures are being currently assessed. Additionally, focusing on the data extracted from the sensor on the lower back might also facilitate the integration of the proposed approach into continuous unsupervised mobility monitoring [25, 46, 54, 55].

Another limitation relates to the results for gait intensity and jerk. Gait velocity was not controlled for, and both people with MS and healthy controls walked at their own comfortable speed. Therefore, our findings for intensity and jerk should be interpreted with caution since they could reflect preferred gait speed [34] and/or movement amplitude and duration [56]. Whilst our protocol was delivered in a clinical environment, it did require six minutes of supervised patient walking which may be hard to replicate in an outpatient clinic setting, and would not be suitable for people at significant risk of falls. Further work will assess whether shorter walking times are able to provide the same amount of reliable information. This might also mitigate any possible limitation associated with fatigue, which we did not assess in this study.

Finally, in view of the relatively small cohort included in this study, we had to group the gait measures into three domains established according to the existing literature [21, 32]. Future studies should include factor analysis to confirm the validity of this decision. While this study was cross-sectional, we are currently longitudinally investigating a larger cohort (MOBILISE-D, www.mobilise-d.eu).

## Conclusions

Our study looked at using small inertial sensors to characterise gait impairment and compensatory strategies in people with progressive MS in their normal clinical setting. This study indicates that people with progressive MS walk at a slower pace, with a much more variable pattern of steps and strides, and experience difficulties in controlling the movements of the trunk and maintaining a stable walk. These abnormalities are more prominent in people with MS with higher levels of disability. These assessments were reliable in test-retest analysis, and suitable for clinical use in monitoring patients and in research settings as accurate and responsive outcome measures for clinical trials.

Ongoing studies will expand on the cross-sectional data presented here and focus on longitudinal observation to assess the responsiveness and validate the use of the proposed gait measures as biomarkers of disease progression within the time course of clinical trials.

## Electronic supplementary material

Below is the link to the electronic supplementary material.

Supplementary file1 (TIF 19685 kb)

Supplementary file2 (DOCX 20 kb)

## Data Availability

Anonymized data can be shared upon reasonable request from other investigators. Researchers can contact Dr. David Paling (David.Paling@nhs.net) to gain access to the data.
