# Supplementary figures and images for: Wearable sensors can reliably quantify gait alterations associated with disability in people with progressive multiple sclerosis in a clinical setting
Source: J Neurol. 2020 May 28;267(10):2897–909. doi: 10.1007/s00415-020-09928-8 (PMC7501113; doi:10.1007/s00415-020-09928-8)

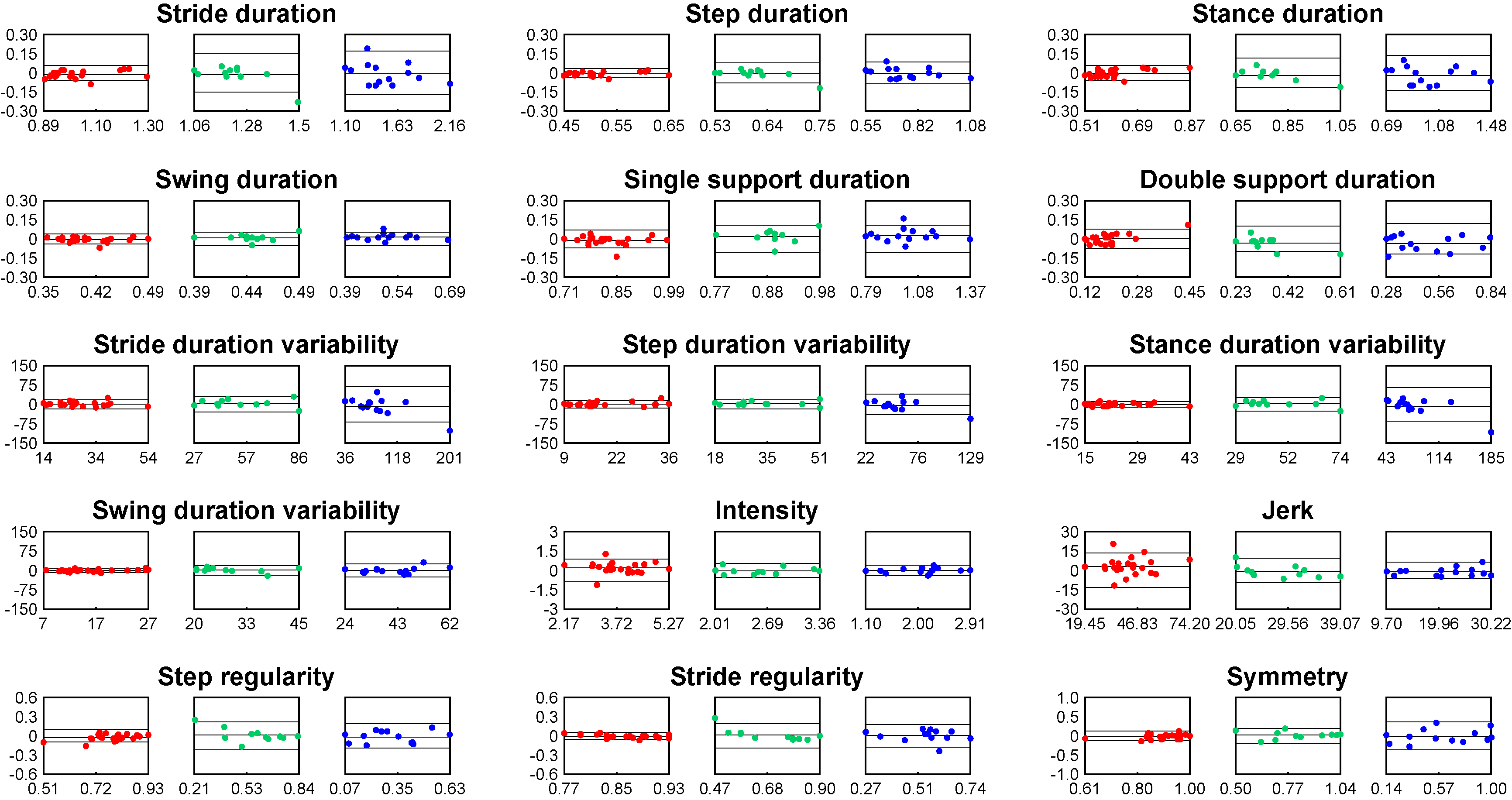

Supplement: Supplementary file 1 — Supplementary file1 (TIF 19685 kb) [file 415_2020_9928_MOESM1_ESM.tif]
