# Supplementary material for: Wearable sensors can reliably quantify gait alterations associated with disability in people with progressive multiple sclerosis in a clinical setting
Source: J Neurol. 2020 May 28;267(10):2897–909. doi: 10.1007/s00415-020-09928-8 (PMC7501113; doi:10.1007/s00415-020-09928-8)
Supplement: Supplementary file 2 — Supplementary file2 (DOCX 20 kb) [file 415_2020_9928_MOESM2_ESM.docx]

**Table 1** Descriptive statistics for the investigated gait measures, together with p-values for the independent Mann-Whitney U Test and associated effect sizes.

|  | ***HEALTHY CONTROLS*** | | | ***MSm GROUP*** | | | ***MSs GROUP*** | | | ***P-VALUE & EFFECT SIZE*** | | |
| --- | --- | --- | --- | --- | --- | --- | --- | --- | --- | --- | --- | --- |
|  | *Median* | *MAD* | *Range* | *Median* | *MAD* | *Range* | *Median* | *MAD* | *Range* | *Ctrl vs MSm* | *Ctrl vs MSs* | *MSm vs MSs* |
| **RHYTHM [s]** |  |  |  |  |  |  |  |  |  |  |  |  |
| *Stride duration* | 0.98 | 0.05 | 0.87–1.28 | 1.19 | 0.07 | 1.00–1.43 | 1.39 | 0.20 | 1.07–3.30 | <.001; -0.6 | <.001; -0.7 | .001; -0.4 |
| *Step duration* | 0.49 | 0.03 | 0.43–0.64 | 0.59 | 0.04 | 0.50–0.71 | 0.70 | 0.10 | 0.53–1.65 | <.001; -0.6 | <.001; -0.7 | .001; -0.4 |
| *Stance duration* | 0.57 | 0.04 | 0.50–0.89 | 0.73 | 0.07 | 0.60–0.97 | 0.87 | 0.12 | 0.62–2.43 | <.001; -0.6 | <.001; -0.7 | .001; -0.5 |
| *Swing duration* | 0.40 | 0.02 | 0.36–0.49 | 0.45 | 0.02 | 0.38–0.52 | 0.51 | 0.06 | 0.36–0.87 | .002; -0.4 | <.001; -0.6 | .007; -0.4 |
| *Single Support duration* | 0.80 | 0.04 | 0.71–0.98 | 0.90 | 0.04 | 0.76–1.04 | 1.01 | 0.11 | 0.72–1.75 | .002; -0.4 | <.001; -0.6 | .006; -0.4 |
| *Double Support duration* | 0.18 | 0.03 | 0.11–0.50 | 0.28 | 0.05 | 0.16–0.55 | 0.38 | 0.09 | 0.16–1.55 | <.001; -0.6 | <.001; -0.7 | .005; -0.4 |
| **VARIABILITY [ms]** |  |  |  |  |  |  |  |  |  |  |  |  |
| *Stride duration* | 26 | 6 | 14–51 | 51 | 11 | 25–115 | 78 | 23 | 39–265 | <.001; -0.7 | <.001; -0.8 | .004; -0.4 |
| *Step duration* | 18 | 6 | 10–46 | 35 | 9 | 17–68 | 44 | 9 | 25–150 | <.001; -0.6 | <.001; -0.7 | .007; -0.4 |
| *Stance duration* | 22 | 5 | 12–39 | 44 | 7 | 24–95 | 67 | 16 | 31–208 | <.001; -0.7 | <.001; -0.8 | .001; -0.4 |
| *Swing duration* | 15 | 5 | 8–31 | 28 | 4 | 15–52 | 38 | 10 | 20–98 | <.001; -0.7 | <.001; -0.7 | .008; -0.4 |
| **BALANCE & COORDINATION** |  |  |  |  |  |  |  |  |  |  |  |  |
| *Intensity [ms^–2^]* | 3.81 | 0.36 | 2.38–5.33 | 2.65 | 0.49 | 1.71–5.30 | 2.30 | 0.43 | 1.08–4.52 | <.001; -0.6 | <.001; -0.7 | .161; -0.2 |
| *Jerk [ms^–3^]* | 44.46 | 7.28 | 20.86–78.35 | 31.28 | 5.83 | 15.03–61.95 | 24.96 | 7.09 | 7.49–45.03 | <.001; -0.5 | <.001; -0.6 | .288; -0.1 |
| *Step Regularity [–]* | 0.78 | 0.05 | 0.46–0.93 | 0.51 | 0.13 | 0.07–0.83 | 0.30 | 0.14 | 0.03–0.64 | <.001; -0.7 | <.001; -0.8 | .005; -0.4 |
| *Stride regularity [–]* | 0.85 | 0.04 | 0.79–0.94 | 0.67 | 0.08 | 0.45–0.90 | 0.58 | 0.08 | 0.15–0.82 | <.001; -0.7 | <.001; -0.8 | .008; -0.4 |
| *Symmetry [–]* | 0.91 | 0.04 | 0.55–0.98 | 0.75 | 0.15 | 0.09–0.94 | 0.57 | 0.24 | 0.06–0.90 | .001; -0.5 | <.001; -0.7 | .03; -0.3 |
